# Standardization of a Method to Evaluate the Antioxidant Capacity of High-Density Lipoproteins

**Published:** 2009-12

**Authors:** Elena de Juan-Franco, Antonio Pérez, Vicent Ribas, Juan Antonio Sánchez-Hernández, Francisco Blanco-Vaca, Jordi Ordóñez-Llanos, José Luis Sánchez-Quesada

**Affiliations:** 1 *Department of Biochemistry, Institut de Recerca, Hospital de la Santa Creu i Sant Pau, Barcelona, Spain*; 2 *Department of Endocrinology, Hospital de la Santa Creu i Sant Pau, Barcelona, Spain*; 3 *CIBER de Diabetes y Enfermedades Metabólicas Asociadas CIBERDEM*; 4 *Department of Biochemistry and Molecular Biology, Universitat Autònoma de Barcelona, Barcelona, Spain*

**Keywords:** HDL, LDL, lipoproteins, HDL antioxidant capacity

## Abstract

Background: A method to evaluate the antioxidant capacity of high-density lipoprotein (HDL) was developed and standardized. Methods: This method measure conjugated diene (CD) formation and electrophoretic mobility of low-density lipoprotein (LDL) in agarose gels in the presence and absence of HDL. HDL was isolated from 1 mL of plasma within 24 hours and oxidation assays were performed within 6 hours. Oxidation was induced by adding CuSO_4_. The lag phase increase in CD kinetics and the inhibition of electrophoretic mobility were defined as the HDL antioxidant capacity. Results: The optimal conditions for the CD assay were 2.5 μM CuSO_4_, LDL at 0.1 g apoB/L, HDL at 0.1 g apoA-I/L, at 37°C and for 3h 50 min. Agarose electrophoresis at 100 V, at 4°C for 50 min was then performed immediately. CD formation variability was 21.1% for inter-assay CV and 12.7% for intra-assay CV. Electrophoretic mobility was 26.5% for inter-assay CV and 2.4% for intra-assay CV. Correlation analysis showed a significant association between the antioxidant capacity of HDL and its neutral/polar lipid ratio. Conclusions: The method herein described measures of the HDL antioxidant capacity in a reproducible and rapid manner that can be applied to a relatively high number of samples.

## INTRODUCTION

Oxidative stress plays a major role in the development of atherosclerotic disease (1). Low-density lipoprotein (LDL) trapped in the arterial intima is oxidized by free radicals generated by surrounding cells, leading to the formation of oxidized LDL (oxLDL). This modified form of LDL promotes inflammatory processes involved in the initiation and development of atherosclerotic plaque, induces cholesterol accumulation in macrophages and causes cytotoxicity and apoptosis in arterial wall cells (1–3). In contrast with LDL, high-density lipoprotein (HDL) has a clearly established antiatherogenic function that is mediated by two independent mechanisms. The first of these is its well-known role in cholesterol reverse transport, removing excess cholesterol from the arterial wall to the feces (4). The second mechanism depends on the inhibition of the oxidative modification of LDL (5, 6). The exact mechanisms by which HDL inhibits LDL oxidation are not well defined but several proteins transported by HDL, such as apolipoprotein A-I (apoA-I), paraoxonase (PON) and platelet-activating factor acetyl-hydrolase (PAF-AH), have been related to its antioxidant properties (7–9). Other physicochemical characteristics of HDL, such as lipid composition, size and density, are supposedly involved in HDL antioxidant capacity (10–12) but their exact role is poorly understood. Furthermore, the few methods available to evaluate these properties are not only complex, cumbersome and of little utility to study a large number of subjects, but moreover, they have not been standardized. The aim of the current work was to develop a reproducible method to measure the HDL capacity to inhibit oxidative modification of LDL.

## METHODS

### Reagents

All reagents were from Sigma-Aldrich (Sigma Espana, Madrid Spain), except for 2-thio-PAF (Cayman Chemicals, Ann Arbor, MI, USA), agarose gels and electrophoresis reagents (Midigel, Biomidi, Toulousse, France), apoB, apoA-I, cholesterol and triglyceride measurement kits (Roche Diagnostics, Basel, Switzerland), and phosholipid and non-esterified fatty acids (NEFA) measurement kits (Wako Chemicals, Neuss, Germany).

### Blood specimens

Lipid profile, PON activity and PAF-AH distribution activity were determined from sera obtained in non-additive vacutainer tubes. Lipoproteins used to measure the antioxidant capacity of HDL were isolated from plasma obtained in EDTA-containing vacutainer tubes. Correlation analysis was performed in 111 plasma samples (53 men, 58 women) consecutively received in our department for routine analysis. The range of plasma lipid concentrations was wide and the sample included normolipemic and hyperlipemic subjects. Those with acute or severe diseases were not included. Samples were assayed for their HDL antioxidant capacity, and lipid and protein HDL composition was determined. The study was approved by the Ethics Committee of the hospital, and volunteers gave their informed consent.

### Lipid profile

The profile of main lipoprotein parameters in sera included total cholesterol (Roche) and triglyceride (Roche), VLDL, LDL and HDL cholesterol, total NEFA (Wako), apoB and apoA-I (Roche) and apoA-II (Kamiya Biomedical, Seattle, WA, USA). Cholesterol of lipoprotein fractions was measured using a direct method to quantify HDL cholesterol (HDL-C plus, Roche), according to NCEP recommendations (13). Apolipoproteins were measured by immunoturbidimetric methods (Roche). All methods were adapted to a Hitachi 911 autoanalyzer.

### Lipoprotein isolation

LDL (density range 1.019–1.063 g/mL) was isolated from a pool of plasma (180 ml) obtained from normolipemic subjects by sequential ultracentrifugation using a preparative fixed-angle rotor (TFT 55.38, Kontron Instruments, Milan, Italy). To avoid lipoperoxidation as far as possible, all solutions contained 1 mmol/L EDTA and 2 μmol/L butylated hydroxytoluene (BHT), and centrifugations were made at 4°C using rotors stored in a cold room. The appropriate density of plasma was obtained by adding solid KBr, as described by Havel *et al* (14). LDL was dialyzed in phosphate buffered saline (PBS) and diluted to 1.1 g apoB/L. Then, 1 mL aliquots were frozen at −80°C in presence of 10% sucrose for a maximum of 8 months. Final LDL concentration in aliquots was 1.0 g apoB/L.

HDL (density range 1.063–1.210 g/mL) was isolated from individual subjects by sequential ultracentrifugation with an analytical fixed-angle rotor (50.3, Beckman Coulter, Fullerton, CA, USA) using 1 mL of plasma. Density solutions needed for isolation of lipoproteins were prepared by adding different amounts of solid KBr to 100 mL of a solution with 200 mmol/L NaCl, 1 mmol/L EDTA and 2 μmol/L BHT (density 1.006 g/mL), according to the equation from Radding and Steinberg (15):

gr of KBr=[volume*(intial denisty−final density)]/[1−(0.312*final density)]

where 0.312 indicates the partial specific volume of KBr at 5°C.

Plasma was adjusted to density 1.063 g/mL by adding 205 μL of a solution of density 1.340 g/mL. After mixing, 1.3 mL of a solution of density 1.063 g/mL was gently superposed and tubes were ultracentrifuged at 50,000 rpm for 8 h at 4°C. VLDL+LDL upper layer was discarded and 1 mL of the infra-natant containing HDL was then adjusted to density 1.210 g/mL by adding 1.11 mL of 1.340 g/mL density solution. After mixing, 0.4 mL of 1.210 g/mL density solution was gently superposed and tubes were ultracentrifuged again at 50,000 rpm for 12 h at 4°C to allow HDL to float and separate from lipoprotein deficient serum.

HDL composition, including total and free cholesterol (Wako), triglycerides, phospholipids (Wako), NEFA, apoA-I, apoA-II and apoB was determined by commercial methods adapted to a Hitachi 911 autoanalyzer (Roche).

### Dialysis of isolated HDL by gel filtration chromatography

Freshly isolated HDL was dialyzed against PBS by gel filtration chromatography using PD-10 Sephadex G25M columns (GE Healthcare, Niscayuna, NY, USA) previously equilibrated with 25 mL of cold PBS. Four hundred μL of HDL were applied to the column and eluted with PBS. The first 3.0 mL were discarded and the next 0.8 mL (corresponding to HDL fractions) were collected. All steps were performed in an ice bath. These volumes differed from those recommended by the manufacturer but were chosen as optimal for dialysis of lipoproteins after several assays (see Results section). ApoA-I content in PBS-dialyzed HDL was measured using a commercial immunoturbidimetric method (Roche) in a Hitachi 911 autoanalyzer, and susceptibility to oxidation assay was immediately performed.

### Conjugated diene formation

**Figure 1:**
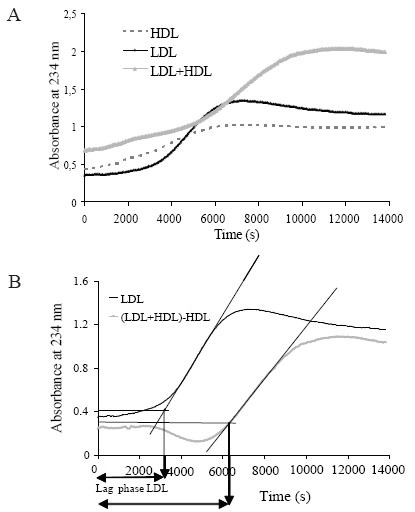
A) Representative oxidation kinetics of LDL (continuous black line), HDL (dashed line) and LDL+LDL (continuous grey line). LDL and HDL (at 0.1 g/L of apoB and apoA-I, respectively) were oxidized in presence of 5 μmol/L CuSO_4_. Lipoproteins were previously dialyzed in PBS; B) Calculation of lag phase time. HDL curve was subtracted from (LDL+HDL) curve, and the curve corresponding to (LDL+HDL)-HDL was obtained. Lag phase time was calculated as shown in the figure and explained in Methods. Maximal slope was considered using 30 consecutive points in the propagation phase.

Oxidation kinetics was monitored by continuously following the formation of conjugated diene, a product of lipid peroxidation with absorbance maximum at 234 nm (16). Kinetics was monitored in 96-microwell plates for UV detection (Greiner, Hannover, Germany) at 234 nm for 3h 50 min and at 37°C, using a Synergy HT multi-detection microplate reader (Bio-Tek, Winooski, VT, USA). The assay was based on the ability of HDL to inhibit LDL oxidation (obtained from a pool of plasma) induced by CuSO_4_. For this purpose, LDL was oxidized alone or in the presence of HDL. In addition, HDL was also oxidized alone. Figure 1A shows typical sigmoid curves of LDL, HDL and HDL+LDL oxidation induced by adding 5 μmol/L CuSO_4_. LDL concentration in all assays was fixed at 0.1 g/L apoB (20 μL of LDL aliquots at 1.0 g/L in a final volume of 200 μL per well). In contrast, several HDL concentrations (0.05, 0.1 and 0.2 g/L apoA-I) and CuSO_4_ (1.5, 2.5, 3.5 and 5 μmol/L) were assayed to achieve the optimal assay conditions. The volume of LDL plus HDL and CuSO_4_ in each well was adjusted to 200 μL with PBS. All measures were performed in duplicate. The goal was to maintain a good balance between the inhibitory effect of HDL and the time of the assay.

The main parameter was lag phase time, calculated from the intersection point between the maximal slope of the curve and initial absorbance, as shown in Figure 1B. The lag phase of the LDL kinetics was considered as 100% oxidation. To evaluate the effect of HDL, the kinetics of HDL alone was subtracted from the kinetics of HDL+LDL to obtain the curve (HDL+LDL)-HDL. As the lag phase of the (HDL+LDL)-HDL curve was longer than that of LDL alone results were expressed as the increment of lag phase time versus LDL alone.

### Agarose gel electrophoresis

Immediately after the kinetics of conjugated diene formation were determined 4 μL of the oxidized samples corresponding to LDL alone and LDL+HDL (but not HDL alone) were subjected to agarose electrophoresis (Lipoprint, Midigel) for 50 min at 100 V in a cold room, using the barbital buffer provided by the manufacturer. A Helena cell chamber (Beaumont, TX, USA) that allows two simultaneous electrophoresis was used. Fixation and staining were also performed as described by the manufacturer. Briefly, gels were fixed with ethanol/acetic acid/water (60:10:30) for 10 minutes, washed with water for 5 minutes and dried under a stream of hot air. Gels were stained for 5 minutes with Sudan Black working solution (methanol/saline/Sudan Black stock solution, 8:4:0.4). Stock solution was prepared dissolving 1 g in 20 mL of dioxane. Finally, gels were destained for 20 min with ethanol/water 50:50 and rinsed twice with water. Each gel contained non-oxidized LDL, oxidized LDL and eight samples with the mixtures of LDL+HDL. Results are expressed as the inhibition of relative mobility (Rf) induced by HDL, considering Rf 0 as the point of sample application and Rf 100 as the mobility of oxidized LDL alone.

### PON activity

Arylesterase activity of PON was quantified from EDTA-free samples using phenylacetic acid as substrate (17). Briefly, 2 μL of serum was incubated with 200 μL of assay buffer (20 mmol/L Tris-HCl, 2 mmol/L CaCl_2_, pH 8.0) containing 3 mmol/L phenylacetic acid (23 μL of phenylacetic acid in 50 mL of assay buffer). Phenylacetic acid was prepared immediately before the assay. With this buffer EDTA-sensitive arylesterase activity, which is due to PON1, was measured. Since other EDTA-resistant arylesterases are present in plasma (18), specific PON1 activity was determined with a parallel measure using the same buffer containing 2 mmol/L EDTA instead of CaCl_2_. Enzymatic kinetics was monitored for 10 min at 25°C at 270 nm (absorbance maximum of phenol, ɛ_270_=1,310 M^−1^ cm^−1^) in 96-multiwell plates for UV detection (Greiner). Activity was calculated from the equation [((Δabs/min) * final vol) / (1,310 * sample vol)] and expressed as μmol/min*mL.

### PAF-AH activity in lipoprotein fractions

PAF-AH activity was measured using 2-thio-PAF (Cayman) as substrate (19), according to the manufacturer’s instructions. Briefly, 200 μL of 2-thio-PAF solution (200 μmol/L in buffer 0.1 mol/L NaCl, 1 mmol/L EGTA, pH 7.2) was added to 10 μL of 5,5′-dithiobis 2-nitrobenzoic acid (DTNB, ɛ_405_=12,800 M^−1^ cm^−1^) (10 mmol/L in Tris-HCl 0.4 mol/L pH 8.0) and 15 μL of sample (serum or precipitated serum). The reaction was monitored at 405 nm for 15 min at 25°C. Activity was calculated from the equation [((Δabs/min) * final vol) / (12,800 * sample vol)] and expressed as μmol/min*mL.

To determine the distribution of PAF-AH between lipoprotein fractions, apoB-containing lipoproteins were precipitated from serum using dextran sulfate (average molecular weight 50 kDa). Two hundred μL of serum was mixed with different volumes (20 μL to 150 μL) of dextran sulfate reagent (0.1 mmol/L dextran sulfate containing 0.25 mmol/L MgCl_2_), incubated for 5 min at room temperature and centrifuged for 10 min at 10,000 g. Two-hundred mL of supernatant (that contains HDL, but not LDL or VLDL) was immediately and carefully collected and assayed for PAF-AH activity. The effect of the triglyceride concentration on the lipoprotein precipitation was studied by assaying different triglyceride levels (from 0.5 to 6.7 mmol/L) and measuring apoB and apoA-I in the supernatant.

### Statistical analysis

The association between the antioxidant capacity of HDL and composition parameters was analyzed by the Spearman correlation coefficient using the SPSS17 statistical package.

## RESULTS

### Lipoprotein dialysis

**Figure 2:**
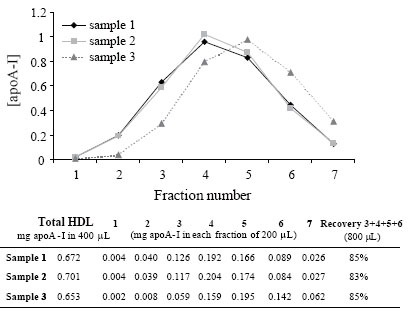
Elution profile of HDL in PD-10 columns. HDL was dialyzed in PBS by gel filtration chromatography using PD-10 columns. Four hundred μL of 3 different HDL samples were fractionated in a PD-10 column, as described in Methods. Seven aliquots of 200 μL were collected, as described in Methods, and apoA-I concentration was measured. The table shows the mg of apoA-I in each aliquot. The recovery of fractions 3 to 6 is indicated in the last column.

Figure 2 shows elution volumes for HDL during PD-10 dialysis. By collecting fractions 3 to 6 (total volume 0.8 mL) we recovered up to 85 % of apoA-I. Dialyzed HDL has a concentration of 0.3–0.6 g/L of apoA-I, depending on the plasma concentration in each subject, sufficient to perform the susceptibility to oxidation assay.

### Optimal HDL and copper concentrations in conjugated diene assay

**Figure 3:**
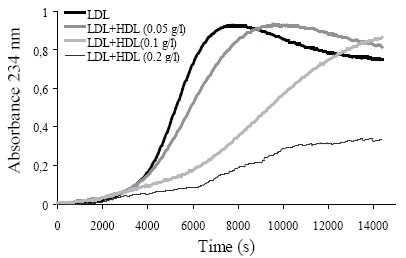
Oxidation of LDL (0.1 g/L apoB) in the presence of increasing HDL concentration (0.05, 0.1 and 0.2 g/L apoA-I). Oxidation was performed at 37°C using 2.5 μM CuSO_4_ with PBS-dialyzed lipoproteins. Kinetics shows LDL alone or LDL+HDL, after subtraction of initial absorbance.

Regarding HDL concentration, Figure 3 shows that a molar ratio of 1:1 between LDL and HDL (expressed as apoB and apoA-I, respectively) was optimal to measure the inhibitory effect of HDL on LDL oxidizability. A higher HDL concentration (0.2 g apoA-I/L) inhibited LDL oxidation excessively and the sigmoid curve was not formed in the assay time. In contrast, we found that a lower HDL concentration (0.05 g apoA-I/L) resulted in a poor inhibitory action.

**Figure 4:**
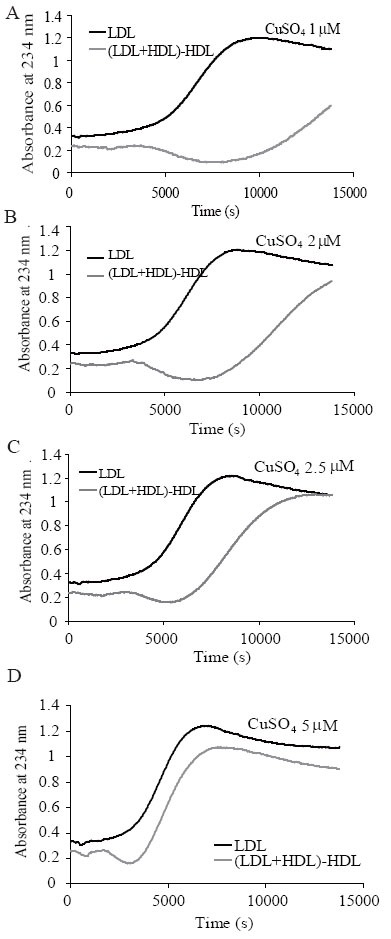
Effect of increasing CuSO_4_ concentration (1, 2, 2.5 and 5 μmol/L) on the conjugated diene kinetics of LDL alone (black lines) and (LDL+HDL)-HDL (grey lines). LDL and HDL were used at 0.1 g/L apoB or apoA-I, respectively. Oxidation was performed at 37°C with PBS-dialyzed lipoproteins.

Figure 4A-D shows representative experiments with varying CuSO_4_ concentrations. When copper concentrations were 1 or 2 μmol/L, a complete sigmoid curve in the assay time was not formed. When the copper concentration used was high (5 μmol/L), the inhibitory effect of HDL (5–30% of lag phase increment) was lower than that observed using 2.5 μmol/L CuSO_4_.

The intra-assay variability of the assay was tested using 4 HDL preparations with different inhibitory capacity (Table 1). The coefficient of variation was lower than 12.7% (n=10) in all HDLs. Regarding inter-assay variability, the coefficient of variation of 7 independent experiments was 21.1% and 15.5%, respectively, in two samples with different inhibitory capacity (Table 1). The relatively high inter-assay variability makes it recommendable to assay related samples, such as those from the same individual before and after treatment, in the same batch.

**Table 1:** Coefficients of variation of the conjugated diene method

n	Sample (HDL)	Mean ± SD (% lag phase increment)	Intra-assay CV (%)

10	1	19.2 ± 2.4	12.7
10	2	64.2 ± 5.7	8.8
10	3	90.5 ± 5.0	5.6
10	4	129.4 ± 12.3	9.5
			**Inter-assay CV (%)**
7	2	64.3 ± 13.5	21.1
7	3	83.0 ± 12.9	15.5

Mean indicates the increment of the lag phase time of LDL kinetic oxidation in the presence of HDL versus the absence of HDL. Four independent HDL samples (numbers 1 to 4) with increasing antioxidant capacity were analyzed for intra-assay variability. Two of these HDL samples (numbers 2 and 3) were analyzed for inter-assay variability.

To test the stability of frozen lipoproteins, 15 independent experiments were performed with the same LDL and HDL preparations, stored at −80°C with sucrose, for a period of 8 months. The inhibition of HDL on LDL oxidizability was 95.5 ± 15.9% (expressed as the increment of lag phase time). The coefficient of variation was 16.7%, similar to that obtained in the inter-assay variability, suggesting that physico-chemical properties of lipoproteins are stable in these storage conditions.

### Agarose gel electrophoresis

**Figure 5:**
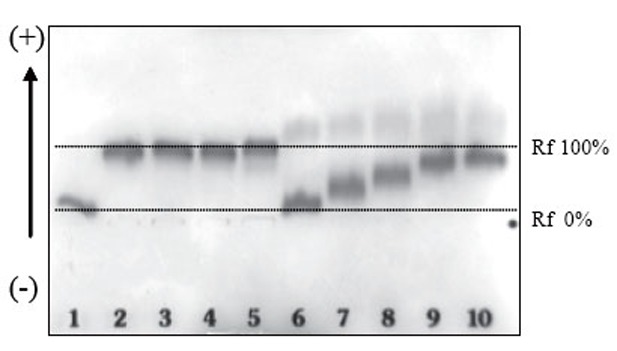
Agarose electrophoresis of LDL alone or LDL+HDL (at 0.1 g/L of apoB or apoA-I, respectively) with increasing amounts of CuSO_4_. Oxidation was performed at 37°C with PBS-dialyzed lipoproteins. Lane 1: native LDL; lane 2: LDL+CuSO_4_ 1 μmol/L; lane 3: LDL+CuSO_4_ 2 μmol/L; lane 4: LDL+CuSO_4_ 2.5 μmol/L; lane 5: LDL+CuSO_4_ 5 μmol/L; lane 6: LDL+HDL+CuSO_4_ 1 μmol/L; lane 7: LDL+ HDL+CuSO_4_ 2 μmol/L; lane 8: LDL+ HDL+CuSO_4_ 2.5 μmol/L; lane 9 and 10: LDL+ HDL+CuSO_4_ 5 μmol/L. Electrophoresis was performed for 50 minutes at 100V, as described in Methods.

The same conditions chosen for conjugated diene formation assay (0.1 g/L of LDL and HDL, 2.5 μM CuSO_4_ and 3h 50min) were used to evaluate the inhibition of the electrophoretic mobility of LDL mediated by HDL. The intra-assay coefficient of variation was lower than 2.5% (n=8, Table 2). Table 2 shows the inter-assay coefficient of variation of 6 independent experiments using two HDL with different antioxidant capacity. Figure 5 shows an agarose gel with LDL or LDL+HDL with different CuSO_4_ concentrations.

**Table 2:** Coefficients of variation using the agarose electrophoresis method

n	Sample (HDL)	Mean ± SD (% Rf inhibition)	Intra-assay CV (%)

8	1	19.2 ± 0.2	1.0
8	2	70.2 ± 1.7	2.4
			**Inter-assay CV (%)**
6	1	15.3 ± 4.1	26.5
6	2	72.4 ± 3.9	5.3

Mean indicates the inhibition of the electrophoretic mobility of LDL kinetic oxidation in the presence of HDL versus the absence of HDL. Two independent HDL samples with different antioxidant capacity were analyzed for intra-assay and inter-assay variability.

### Relation of the antioxidant capacity of HDL with its protein and lipid composition

In order to determine the main determinants of the antioxidant capacity of HDL in the general population, plasma samples from 111 subjects that arrived consecutively to our laboratory for routine analysis were assayed for their HDL antioxidant capacity, HDL composition, PON1 activity and HDL-bound PAF-AH activity. Some HDL preparations from hypertriglyceridemic subjects had an abnormally high content of triglycerides (higher than 9% of lipoprotein mass) and presence of detectable apoB. This observation indicated possible contamination with VLDL. Hence, samples with detectable apoB and high triglyceride content were excluded from the analysis. The results of the correlation analysis (n=96) are shown in Table 3. Both methods to measure the antioxidant capacity showed a significant correlation (r=0.294, P<0.004). However, the correlation with other compositional parameters of HDL was different. The inhibition of electrophoretic mobility only showed positive correlation with NEFA in HDL (Table 3). This indicates that a high NEFA concentration could disturb results obtained by agarose electrophoresis.

**Table 3:** Spearman correlation coefficients between the antioxidant capacity of HDL and parameters of HDL composition (n=96)

	% of lag phase increment		% of Rf inhibition	
	R	P	R	P

Esterified cholesterol (%)	0.198	0.053	−0,195	0.055
Free cholesterol (%)	0.239	0.019^d^	−0.118	0.248
Triglyceride (%)	0.152	0.140	0.154	0.133
Phospholipid (%)	−0.021	0.428	0.165	0.106
NEFA (mol/mol apoA-I)	0.146	0.157	0.301	0.003^d^
apoA-I (%)	−0,201	0.050^d^	−0.126	0.220
apoA-II (%)	−0.035	0.734	0.013	0.902
PON1 (plasma activity)	−0.058	0.577	0.108	0.297
PAF-AH (HDL activity)	0.097	0.349	−0.023	0.822
Neutral lipids/polar lipids^a^	0.314	0.002^d^	−0.166	0.105
Core/surface^b^	0.366	<0.001^d^	−0.135	0.189
Lipid/protein^c^	0.150	0.144	0.086	0.403

a Ratio triglycerides+esterified cholesterol/phospholipids+free cholesterol;

b Ratio triglycerides + esterified cholesterol/phospholipids + free cholesterol+apoA-I+apoA-II;

c Ratio triglycerides+cholesterol+phospholipids/apoA-I+apoA-II;

d Statistically significant associations.

Regarding the increase of lag phase, free cholesterol content in HDL was positively associated with the protective action of HDL whereas apo-AI content showed a mild negative correlation with such an increase (Table 3). The neutral/polar lipids and core/surface ratios presented strong positive correlations with the increment of lag phase time. Taken together these results indicate that the higher the neutral lipids content and the core material are the higher antioxidant capacity of HDL.

## DISCUSSION

Several methods have been described for the ultra-rapid isolation of lipoprotein fractions. The combination of density gradient ultracentrifugation with vertical or near-vertical rotors separates lipoproteins very quickly but isolated lipoprotein fractions are not sufficiently pure for experiments to evaluate the antioxidant properties of HDL (20). Fixed-angle rotors and sequential ultracentrifugation have the advantage of fewer contaminating proteins in isolated lipoprotein fractions (20). Several methods to isolate lipoproteins by sequential ultracentrifugation in only 6–8 hours have been described (21) but the use of very high centrifuge forces (higher than 500,000 g) could result in partial loss of proteins responsible for the qualitative properties of lipoproteins, such as LCAT, PAF-AH or PON. We chose to use a lower centrifugal force (200,000 g) and two sequential separations that permitted isolation of VLDL+LDL on the first working day in a first step, and isolation of HDL overnight, in a second step. A critical step for the oxidation assay is the elimination of KBr, EDTA, BHT and other antioxidant components present in buffers and density solutions. Most authors use extensive dialysis for more than 24 h at 4°C. However, this could initiate oxidation of lipoproteins before the assay is performed. To avoid this possibility, in the present study we used gel filtration chromatography in PD10 columns.

One major drawback of several assays that measure absorbance at 234 nm is that the contribution of HDL lipids to conjugated diene kinetics is not taken into account (12, 22). Lipids in HDL oxidize simultaneously with lipids in LDL. Hence, the HDL kinetic curve must be subtracted from LDL+HDL kinetics. The optimal conditions for the CD assay were 2.5 μM CuSO_4_, LDL at 0.1 g apoB/L, HDL at 0.1 g apoA-I/L, at 37°C and for 3h 50 min. In keeping with our findings, LDL/HDL ratios of 1:2 (expressed as total protein) have been reported to increase lag phase by approximately 300% (23). In contrast, lower HDL concentration (0.05 g apoA-I/L) resulted in a poor inhibitory action. Further in the favor of a molar ratio of 1:1 between LDL and HDL is that it is similar to the ratio usually present in plasma, and represents an experimental condition closer to the physiologic environment. Regarding CuSO_4_ concentration, previous studies reported poor protective action of HDL when the oxidative stimulus is strong (12). Thus, 2.5 μmol/L CuSO_4_ was chosen as the optimal concentration. The stability of frozen lipoproteins, stored at −80°C with sucrose, was good up to 8 months. It has been previously reported that freezing with sucrose preserves most physico-chemical properties of lipoproteins (24).

The results of the correlation analysis indicated that a high NEFA concentration could disturb results obtained by agarose electrophoresis. The disturbing effect is probably due to the high electronegative charge that NEFA confers to lipoproteins, modifying their electrophoretic behavior. The conjugated diene method should therefore be used when a high NEFA concentration is suspected, such as in poorly controlled diabetic patients, obese subjects, or in postprandial samples.

Regarding the increase of lag phase, our results indicate that the higher the neutral lipids content and the core material are the higher antioxidant capacity of HDL. Accordingly, HDL with higher lipid content had enhanced ability to lengthen the oxidation kinetics of LDL. These results are in contrast with previous reports that small dense HDL is more protective than large buoyant HDL (12, 22). This difference could be attributed to the fact that we equaled HDL by its apoA-I content whereas those authors equaled HDL by its cholesterol content. The number of cholesterol molecules per HDL particle (free and esterified) varies from 200–250 in HDL2 to 50–160 in HDL3 (25). Since the cholesterol content is much lower in small HDL than in large HDL, more HDL particles per LDL particle are added to the assay mixture when small HDL is used.

In our opinion, the right way to equal HDL concentration would be the number of particles in the assay tube. However, at the present day the only method is NMR but this is not available for most laboratories. Thus, apoA-I concentration gives a more accurate measure of the number of HDL particles than cholesterol since cholesterol is the most variable lipid in HDL due to its role in reverse cholesterol transport. On the other hand, free cholesterol was positively associated with the increase of the lag phase, in agreement with reports showing that high free cholesterol content increases the resistance to oxidation of lipoproteins (26) and that free cholesterol is increased in large HDL particles (25).

Further discussion is needed concerning the lack of statistical correlation of PON1 and PAF-AH activities with the HDL antioxidant capacity. The antioxidant properties mediated by the peroxidase activity of PON1 are well-established (5, 7, 12, 24) but recent findings have raised some doubts regarding the relative contribution of this enzyme to the antioxidant capacity of HDL. PON1 is a calcium-dependent enzyme and EDTA abolishes its antioxidant ability. Kontush *et al* reported that PON1 activity measured in EDTA-plasma samples dramatically decreased to 0.1–5% compared with serum samples, but the antioxidant capacity of HDL was similar in both types of sample (12). Despite this observation, these authors reported an association between PON1 activity and HDL antioxidant capacity (12, 24). However, other authors have not found this association (27, 28) and evidence for a PON-independent inhibition of LDL oxidation by HDL has been reported (28). Our results suggest that the lack of correlation between PON1, measured in serum, and the antioxidant capacity of HDL, isolated from EDTA-plasma, could be related with the inactivation of PON1 in isolated HDL.

Regarding PAF-AH activity, this enzyme hydrolyzes oxidized fractionated phospholipids (also named PAF-like phospholipids) to form lysophosphatidylcholine and oxidized short-chain fatty acids (29). Thus, PAF-AH plays a major role in the inhibition of the high inflammatory potential of PAF-like phospholipids (30). However, it is not clear whether PAF-AH plays a direct role in the inhibition of LDL oxidation. Even though PAF-AH could hydrolyze oxidized phospholipids formed throughout the oxidative process, oxidized short-chain fatty acids remain in the LDL+HDL mixture. It should be taken into account that human PAF-AH is also present in LDL (8, 19, 30).

In summary, the method herein described measures of the HDL antioxidant capacity in a reproducible and relatively rapid manner. This standardized method can be applied to a relatively high number of samples. HDL is isolated in 24 h and oxidation analysis can be performed in one working day. Agarose electrophoresis is not recommended, however, due to the possible interference of a high plasma NEFA concentration. Our results suggest that the main determinant of the antioxidant capacity of HDL is its content in neutral lipids.
